# Burden of vision loss in the Eastern Mediterranean region, 1990–2015: findings from the Global Burden of Disease 2015 study

**DOI:** 10.1007/s00038-017-1000-7

**Published:** 2017-08-03

**Authors:** GBD 2015 Eastern Mediterranean Region Vision Loss Collaborators, Sare Safi, Hamid Ahmadieh, Marzieh Katibeh, Mehdi Yaseri, Alireza Ramezani, Saeid Shahraz, Maziar Moradi-Lakeh, Ibrahim Khalil, Charbel El Bcheraoui, Michael Collison, Adrienne Chew, Farah Daoud, Kristopher J. Krohn, Zane Rankin, Ashkan Afshin, Nicholas J. Kassebaum, Helen E. Olsen, Jeffrey D. Stanaway, Haidong Wang, Katie Wilson, Gebre Yitayih Abyu, Ayman Al-Eyadhy, Khurshid Alam, Deena Alasfoor, Reza Alizadeh-Navaei, Rajaa Al-Raddadi, Ubai Alsharif, Khalid A. Altirkawi, Nahla Anber, Hossein Ansari, Palwasha Anwari, Hamid Asayesh, Solomon Weldegebreal Asgedom, Tesfay Mehari Atey, Umar Bacha, Aleksandra Barac, Neeraj Bedi, Zahid A. Butt, Abdulaal A. Chitheer, Shirin Djalalinia, Huyen Do Phuc, Babak Eshrati, Maryam S. Farvid, Farshad Farzadfar, Seyed-Mohammad Fereshtehnejad, Florian Fischer, Tsegaye Tewelde Gebrehiwot, Randah Ribhi Hamadeh, Samer Hamidi, Tarig B. Higazi, Mohamed Hsairi, Aida Jimenez-Corona, Denny John, Jost B. Jonas, Amir Kasaeian, Yousef Saleh Khader, Ejaz Ahmad Khan, Heidi J. Larson, Asma Abdul Latif, Raimundas Lunevicius, Hassan Magdy Abd El Razek, Mohammed Magdy Abd El Razek, Azeem Majeed, Reza Malekzadeh, Colm McAlinden, Ziad A. Memish, Ted R. Miller, Seyed-Farzad Mohammadi, Vinay Nangia, Cuong Tat Nguyen, Quyen Le Nguyen, Felix Akpojene Ogbo, Farshad Pourmalek, Mostafa Qorbani, Anwar Rafay, Vafa Rahimi-Movaghar, Rajesh Kumar Rai, Saleem M. Rana, David Laith Rawaf, Salman Rawaf, Andre M. N. Renzaho, Satar Rezaei, Gholamreza Roshandel, Mahdi Safdarian, Saeid Safiri, Payman Salamati, Abdallah M. Samy, Benn Sartorius, Sadaf G. Sepanlou, Masood Ali Shaikh, Eirini Skiadaresi, Badr H. A. Sobaih, Rizwan Suliankatchi Abdulkader, Hugh R. Taylor, Arash Tehrani-Banihashemi, Mohamad-Hani Temsah, Roman Topor-Madry, Bach Xuan Tran, Miltiadis Tsilimbaris, Kingsley Nnanna Ukwaja, Olalekan A. Uthman, Tolassa Wakayo, Naohiro Yonemoto, Mustafa Z. Younis, Maysaa El Sayed Zaki, Aisha O. Jumaan, Theo Vos, Simon I. Hay, Mohsen Naghavi, Christopher J. L. Murray, Ali H. Mokdad

**Affiliations:** Institute for Health Metrics and Evaluation, University of Washington, Seattle, WA USA

**Keywords:** Eastern Mediterranean region, Global burden of disease, Vision impairment, Vision disorder

## Abstract

**Objectives:**

To report the estimated trend in prevalence and years lived with disability (YLDs) due to vision loss (VL) in the Eastern Mediterranean region (EMR) from 1990 to 2015.

**Methods:**

The estimated trends in age-standardized prevalence and the YLDs rate due to VL in 22 EMR countries were extracted from the Global Burden of Disease (GBD) 2015 study. The association of Socio-demographic Index (SDI) with changes in prevalence and YLDs of VL was evaluated using a multilevel mixed model.

**Results:**

The age-standardized prevalence of VL in the EMR was 18.2% in 1990 and 15.5% in 2015. The total age-standardized YLDs rate attributed to all-cause VL in EMR was 536.9 per 100,000 population in 1990 and 482.3 per 100,000 population in 2015. For each 0.1 unit increase in SDI, the age-standardized prevalence and YLDs rate of VL showed a reduction of 1.5% (*p* < 0.001) and 23.9 per 100,000 population (*p* < 0.001), respectively.

**Conclusions:**

The burden of VL is high in the EMR; however, it shows a descending trend over the past 25 years. EMR countries need to establish comprehensive eye care programs in their health care systems.

**Electronic supplementary material:**

The online version of this article (doi:10.1007/s00038-017-1000-7) contains supplementary material, which is available to authorized users.

## Introduction

Vision loss is an important public health issue worldwide. About 90% of people with visual impairment live in developing countries (Congdon et al. 2003; Tabbara 2001; WHO 2004, 2013). According to the Global Burden of Disease (GBD) 2015 study, 34.3 million people are blind globally. In addition, 214 million and 24.3 million people suffer from moderate and severe vision impairment (VI), respectively. Vision loss was the third-ranked impairment after anemia and hearing loss in the GBD 2015 study (GBD 2015). Vision loss affects the quality of life of the affected individuals and their families, increases the risk of death by raising the risk of accidents, and increases the financial burden (McCarty et al. 2001; Taylor et al. 1991, 2006). The World Health Organization (WHO) Global Action Plan 2014–2019 emphasized the importance of collecting data on the burden and causes of VI. Periodic studies were recommended to identify the burden of vision loss and the avoidable causes of VI and blindness to achieve a Global Action Plan target and plan health policies (WHO 2013).

Uncorrected refractive errors (RE), cataract, glaucoma, age-related macular degeneration (AMD), diabetic retinopathy (DR), trachoma, and corneal opacities were the main causes of global VI reported by WHO in 2010 (Pascolini and Mariotti 2012). Uncorrected RE, cataract, and glaucoma accounted for 43, 33, and 2% of VI, respectively (Pascolini and Mariotti 2012). Hence, almost 80% of VI is preventable or treatable, and cost-effective interventions can decrease the burden of VI (WHO 2013).

The Eastern Mediterranean region (EMR) has a population of about 583 million and consists of 22 countries (WHO 2017). These countries are not uniform in terms of lifestyle, gross domestic product, and socioeconomic status (Mandil et al. 2013; Mokdad et al. 2014). A previous study reported a descending trend in age-standardized prevalence of blindness (from 2.1 to 1.1%) and moderate and severe VI (from 7.1 to 4.5%) in this region in 2010 (Khairallah et al. 2014). Despite that, the EMR is one of four regions with a greater than 4% prevalence of blindness among older adults (≥50 years), compared to ≤0.4% in high-income regions (Stevens et al. 2013). The current study aims to present trends in prevalence and years lived with disability (YLDs) rate for the main causes of vision loss in EMR countries by sex and age from 1990 to 2015 using the results of the GBD 2015 study. Considering the diversity of the EMR countries in terms of socioeconomic status, the relationship between the prevalence of vision loss and Socio-demographic Index (SDI) was also evaluated.

## Methods

We used data from the Global Burden of Disease 2015 study (GBD 2015). The methodology of the GBD 2015 study for estimating the prevalence of vision loss has been comprehensively described in a recent GBD publication (GBD 2015). In brief, the GBD group estimated the prevalence of vision loss by defining VI and blindness, providing the input model, and defining the modeling strategy. VI was defined as visual acuity (VA) <6/18 based on the Snellen chart, while the blindness definition was VA <3/60 or visual field around central fixation <10%. Uncorrected RE, cataract, glaucoma, macular degeneration, and other causes including DR, trachoma, vitamin A deficiency, retinopathy of prematurity (ROP), meningitis, encephalitis, and onchocerciasis were used for modeling vision loss (GBD 2015).

The vision loss data were from population‐based studies that measured VA. Both peer‐reviewed publications and gray literature were used. Those which reported the causes of vision loss were used for estimating the VI and blindness prevalence due to cataract, glaucoma, macular degeneration, DR, and other causes (GBD 2015). Studies missing best-corrected or presenting VA were excluded (GBD 2015).

A systematic literature review was performed for the period of January 1, 2013–May 20, 2015, to add new evidence to that compiled for GBD 2013. Additionally, the data were extracted from WHO-sponsored Studies on Global Ageing and Adult Health (SAGE) and the United States National Health and Examination Surveys (NHANES) as the nationally representative reviews that measured VA. In addition to SAGE and NHANES, the Surveys of Health, Ageing, and Retirement in Europe (SHARE); the Multi‐Country Survey Study on Health and Responsiveness (MCSS); and the World Health Surveys (WHS) studies were assessed to extract the data with self-reported near VA (GBD 2015).

The prevalence of vision loss was modeled in three stages. At first, the prevalence of moderate and severe VI, blindness, and presbyopia was evaluated to calculate the total presenting vision loss estimation. Secondly, the proportion of presenting vision loss attributed to uncorrected RE was estimated. Thirdly, the prevalence of vision loss due to cataract, glaucoma, macular degeneration, DR, ROP, trachoma, vitamin A deficiency, onchocerciasis, meningitis, and other causes was assessed (GBD 2015).

The YLDs rate, a GBD metric, demonstrates years lived in less than ideal health and is calculated by sequela as prevalence multiplied by the disability weight for the condition associated with that sequela (GBD 2015).

We extracted the estimated trends in prevalence and YLDs rates for vision loss and four leading causes, including refraction and accommodation disorders, cataract, glaucoma, and macular degeneration, in Afghanistan, Bahrain, Djibouti, Egypt, Iran, Iraq, Jordan, Kuwait, Lebanon, Libya, Morocco, Oman, Pakistan, Palestine, Qatar, Saudi Arabia, Somalia, Sudan, Syria, Tunisia, the United Arab Emirates, and Yemen from 1990 to 2015 from the GBD 2015 study using an online interactive tool (https://vizhub.healthdata.org/gbd-compare/) developed by the Institute for Health Metrics and Evaluation. We also determined the relationship between SDI and the reduction in prevalence of vision loss and YLDs rate. The SDI was developed for GBD 2015 to provide an interpretable synthesis of overall development, as measured by lag-dependent income per capita, average educational attainment in the population over 15 years, and the total fertility rates. In GBD 2015, SDI was computed by rescaling each component to the scale of zero to one, and then taking the geometric mean of these values for each location-year. Zero indicates the lowest observed educational attainment, lowest income per capita, and highest fertility rate from 1980 to 2015, and one indicates the highest observed educational attainment, highest income per capita, and lowest fertility rate during that time (GBD 2015).

### Statistical methods

A multilevel linear model was used to assess the relation of SDI values extracted from the GBD 2015 study for 22 countries in the EMR and the changes in prevalence and YLDs during the period 1990–2015. In this way, the probable geographic correlation was also considered. All statistics were presented with 95% uncertainty intervals (95% UI). Statistical analysis was performed using lme4 package in R software (version 3.2.3) (Bates et al. 2015).

## Results

**Fig. 1:**
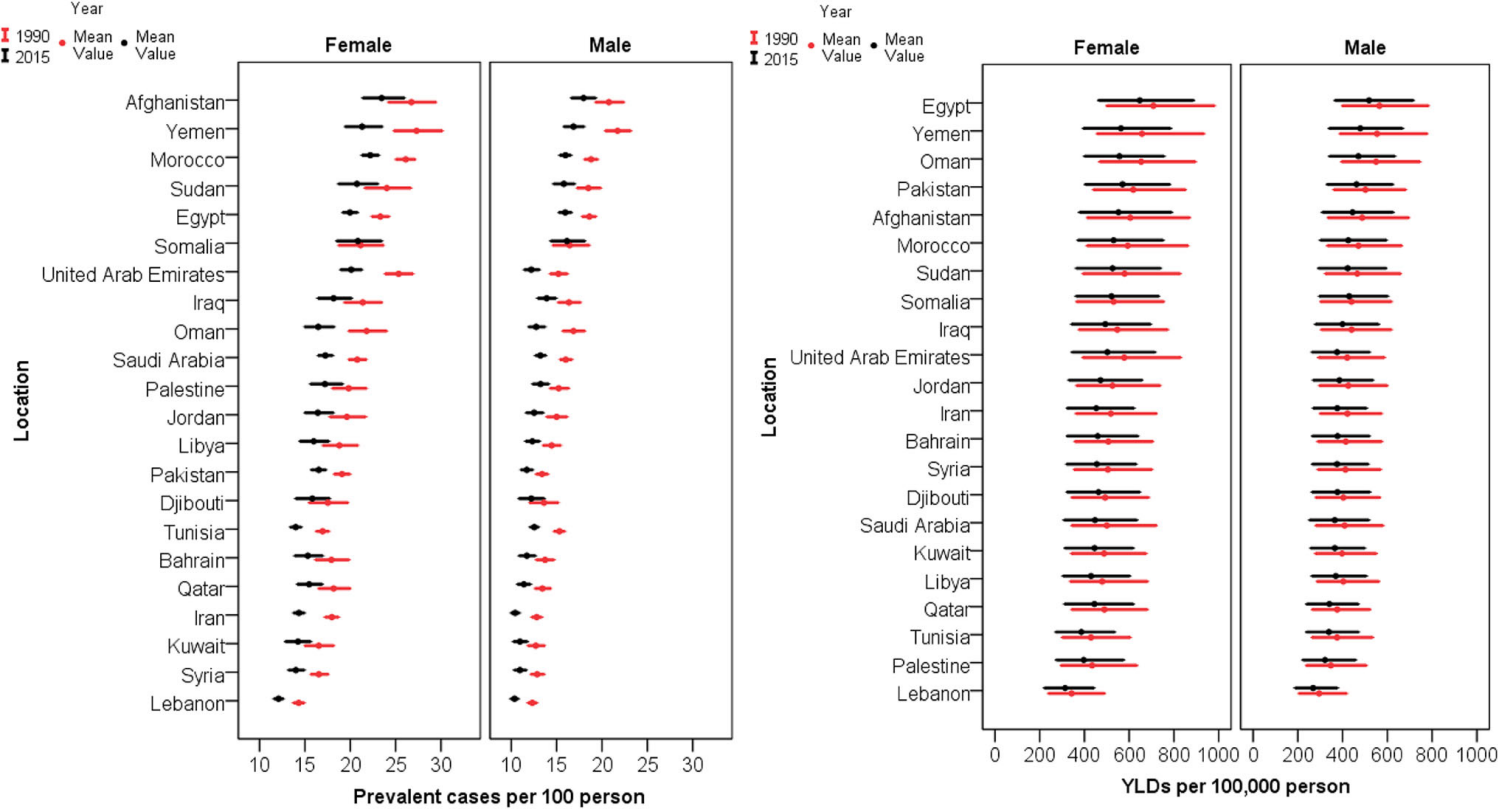
Age-standardized prevalence and years lived with disability (YLDs) rate of vision loss in Eastern Mediterranean Region countries in 1990 and 2015 (Global Burden of Disease Study 2015, Eastern Mediterranean Countries, 1990–2015)

The age-standardized prevalence of vision loss in the EMR was 18.2% (95% UI 17.5–19%) in 1990 and 15.5% (95% UI 14.8–16.2%) in 2015. The total age-standardized YLDs rate attributed to vision loss in EMR was 536.9 per 100,000 population (95% UI 378.5–746) in 1990 and 482.3 per 100,000 population (95% UI 342.5–667.8) in 2015. Vision loss was more prevalent in females than males in both 1990 and 2015 (*p* < 0.001). There were similar findings in terms of the estimated YLDs rate (*p* < 0.001) (Fig. 1).

Table 1 shows decreased prevalence of vision loss and the attributed YLDs rate in all 22 countries from 1990 to 2015. The highest decrease was observed in Oman (prevalence −26.6, YLDs −16.2), while the lowest was observed in Somalia (prevalence −1.6, YLDs −2.0).

**Table 1:** Age-standardized prevalence and years lived with disability rate per 100,000 population of vision loss in the Eastern Mediterranean Region in 1990 and 2015 (Global Burden of Disease Study 2015, Eastern Mediterranean Countries, 1990–2015)

Location	Prevalence (%)						Percent change	YLDs per 100,000 population						Percent change
	1990			2015				1990			2015			
	Value	95% UI		Value	95% UI			Value	95% UI		Value	95% UI		
		Lower	Upper		Lower	Upper			Lower	Upper		Lower	Upper	
Eastern Mediterranean Region	18.2	17.5	19	15.5	14.8	16.2	−15.1	536.9	378.5	746	482.3	342.5	667.8	−10.2
Afghanistan	23.8	21.9	25.8	20.7	19.1	22.4	−12.9	547.1	374.5	785.5	499	341.9	711.6	−8.8
Bahrain	15.5	14.3	16.9	13.2	12.2	14.4	−14.7	454.2	318.6	631.1	412.6	290.3	573.3	−9.2
Djibouti	15.6	13.9	17.5	14.1	12.5	15.7	−10.2	450.2	312.3	627.6	421.1	291.2	587.5	−6.5
Egypt	21.1	20.3	21.9	18.1	17.3	18.8	−14.5	642.7	456.6	893.5	586.8	418.2	807.3	−8.7
Iran	15.3	14.7	15.9	12.3	11.8	12.9	−19.5	468.3	331.2	647	413.2	295.4	563.9	−11.8
Iraq	18.9	17.4	20.6	16.1	14.8	17.6	−14.7	495.3	339.9	697	449.2	312.2	636.2	−9.3
Jordan	17.3	15.9	18.7	14.5	13.3	15.8	−16.1	473.9	329.1	667.6	429	299.7	596	−9.5
Kuwait	14.3	13.3	15.5	12.4	11.4	13.4	−13.7	436.2	305.6	604.9	398.8	280.1	552.1	−8.6
Lebanon	13.3	12.8	13.9	11.2	10.7	11.6	−16.1	319.4	220.9	457.4	290.6	202.7	408.5	−9
Libya	16.5	15.3	18	14.2	13.2	15.4	−14	439.8	310.3	613.3	400.3	283.8	556.6	−9
Morocco	22.7	21.9	23.5	19.2	18.5	19.9	−15.3	536.5	373.2	770.7	480.1	335.1	677.7	−10.5
Oman	19.3	17.9	20.8	14.1	13.2	15.3	−26.6	602.4	434.9	824.1	505	364.4	683.1	−16.2
Pakistan	16	15.3	16.8	14.1	13.4	14.8	−12.3	555.3	395.6	760.1	514.7	368.2	699	−7.3
Palestine	17.6	16.3	19	15.3	14.1	16.5	−13.3	392.7	267.6	574.1	360.4	249.3	517.5	−8.2
Qatar	15	14	16.1	12.6	11.8	13.5	−15.7	414.5	290.7	577.6	373.3	261.2	515.5	−9.9
Saudi Arabia	18.2	17.5	18.9	15	14.4	15.7	−17.4	451.8	310.1	644.2	402.3	275.8	569.2	−11
Somalia	18.9	16.8	21.2	18.6	16.6	20.8	−1.6	487.1	335.3	690.9	477.2	329.9	671	−2
Sudan	21.3	19.7	23.1	18.3	16.8	20	−14.1	524.5	360.6	744.2	476.1	327.7	669.7	−9.2
Syria	14.7	13.9	15.6	12.5	11.8	13.4	−14.7	458.8	321.9	637.8	416.7	294.7	574.1	−9.2
Tunisia	16.1	15.5	16.7	13.3	12.8	13.8	−17.6	402.3	280.7	571.8	364	254.4	506	−9.5
United Arab Emirates	18.8	17.7	19.9	14.2	13.4	15.2	−24.4	478.5	329	678.5	408.6	284.8	572.7	−14.6
Yemen	24.8	23	26.8	19.2	17.8	20.7	−22.8	611.7	425.3	870.5	522.5	367.4	726.7	−14.6

*UI* uncertainty interval, *YLDs* years lived with disability

**Fig. 2:**
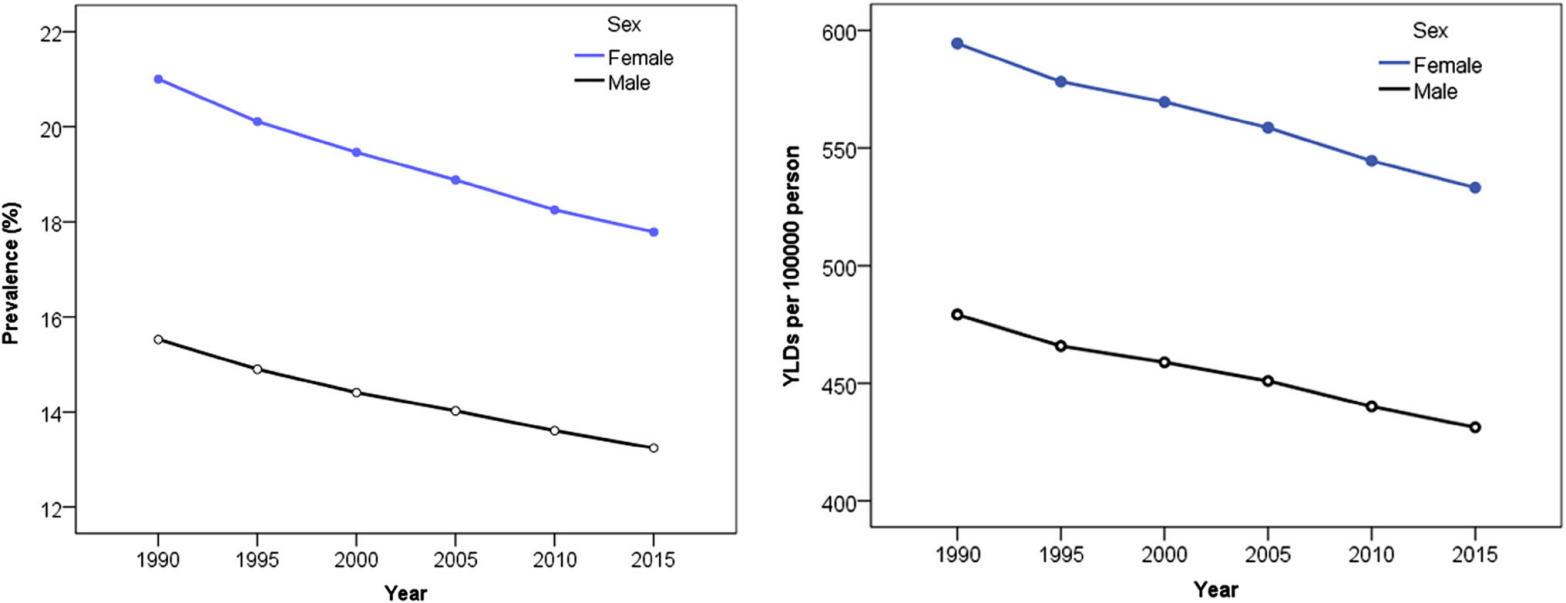
Trends in age-standardized prevalence and years lived with disability (YLDs) rate of vision loss by gender in the Eastern Mediterranean Region from 1990 to 2015 (Global Burden of Disease Study 2015, Eastern Mediterranean Region, 1990–2015)

A descending trend in the age-standardized prevalence and YLDs rates for vision loss was noted among both sexes from 1990 to 2015 (Fig. 2).

The age-standardized prevalence and YLDs rates of vision loss in the EMR were higher than the global rate and ranked third following the Southeast Asia and Africa regions in 1990 and 2015. However, this region had the highest reduction from 1990 to 2015 compared to all six world regions (ESM_1).

### Age-standardized prevalence and YLDs for causes of vision loss

#### Refraction and accommodation disorders

The total age-standardized prevalence of refraction and accommodation disorders in the EMR was 12.9% (95% UI 12.4–13.4%) in males and 17.8% (95% UI 17.1–18.6%) in females in 1990. In 2015, these values were 10.8% (95% UI 10.3–11.2%) in males and 14.8% (95% UI 14.1–15.5%) in females. The prevalence of this disorder in 1990 was highest in Yemen and lowest in Lebanon. In 2015, Afghanistan had the highest prevalence in terms of refraction and accommodation disorders (ESM_2). The age-standardized YLD rate from refraction and accommodation disorders in the EMR in 1990 amounted to 263.3 (95% UI 174.3–395.6) per 100,000 males and 337.9 (95% UI 221.9–518.7) per 100,000 females. In 2015, YLDs attributed to refraction and accommodation disorders in the EMR were 231.9 (95% UI 154.4–344.7) per 100,000 males and 296.4 (95% UI 195.4–446.4) per 100,000 females. In 1990 and 2015, Egypt had the highest age-standardized YLDs per 100,000 person, and Lebanon had the lowest (ESM_2).

#### Cataract

The age-standardized prevalence of cataract in the EMR was 1.5% (95% UI 1.3–1.6%) among males and 1.8% (95% UI 1.6–2.0%) among females in 1990. The corresponding values were 1.3% (95% UI 1.2–1.5%) in males and 1.7% (95% UI 1.5–1.9%) in females in 2015 (ESM_3). Cataract was most prevalent in Pakistan and least prevalent in Libya in both 1990 and 2015.

Age-standardized YLDs attributed to cataract in the EMR in 1990 were 97.9 (95% UI 70.3–132.7) per 100,000 population in males and 124.4 (95% UI 89.0–168.3) per 100,000 population in females. In 2015, the corresponding values were 84.9 (95% UI 60.6–113.9) per 100,000 population in males and 113.6 (95% UI 81.6–152.5) per 100,000 population in females (ESM_3). The highest rate of YLDs per 100,000 population for cataract were observed in Pakistan, and the lowest in Lebanon both in 1990 and 2015.

#### Glaucoma

In both 1990 and 2015, the highest age-standardized prevalence of glaucoma was observed in Egypt, and the lowest was reported in Afghanistan. Total age-standardized prevalence of glaucoma in the EMR was 0.1% (95% UI 0.1–0.1%) in males and 0.2% (95% UI 0.1–0.2%) in females in 1990, and 0.1% (95% UI 0.1–0.1%) in males and 0.2% (95% UI 0.1–0.2%) in females in 2015 (ESM_4).

In 1990, glaucoma accounted for 10.7 age-standardized YLDs (95% UI 7.3–14.8) per 100,000 for males and 15.4 age-standardized YLDs (95% UI 10.5–21.1) per 100,000 for females in the EMR. The rate of age-standardized YLDs for glaucoma in 2015 was 11.5 (95% UI 7.9–16.1) per 100,000 for males and 16.7 (95% UI 11.6–23.1) per 100,000 females in this region (ESM_4). YLDs attributed to glaucoma were highest in Oman and lowest in Pakistan in 1990, and highest in Egypt and lowest in Lebanon in 2015.

#### Macular degeneration

The age-standardized prevalence of macular degeneration in the EMR was 0.1% (95% UI 0.1–0.1%) among males and 0.1% (95% UI 0.1–0.1%) among females in 1990. In 2015, the corresponding values were 0.1% (0.1–0.1%) in males and 0.1% (95% UI 0.1–0.1%) in females in 2015 (ESM_5). Among the 22 countries, Kuwait had the highest and Afghanistan had the lowest prevalence of macular degeneration in 1990. However, macular degeneration was most prevalent in Oman and least prevalent in Somalia in 2015.

The age-standardized YLDs rate associated with macular degeneration in the EMR was 6.0 (95% UI 4.1–8.3) per 100,000 for males and 6.5 (95% UI 4.5–9.1) per 100,000 for females in 1990, increasing to 8.1 (95% UI 5.6–11.2) per 100,000 males and 9.0 (95% UI 6.2–12.6) per 100,000 females in 2015 (ESM_5). In both 1990 and 2015, Oman had the highest and Somalia had the lowest YLDs attributed to macular degeneration.

#### Other causes of vision loss

Saudi Arabia had the highest prevalence of other causes of vision loss, and Somalia had the lowest. In 1990, the age-standardized prevalence of other causes of vision loss was 0.4% (95% UI 0.4–0.4%) in males and 0.5% (95% UI 0.4–0.5%) in females. Corresponding values were 0.5% (95% UI 0.4–0.5%) in males and 0.5% (95% UI 0.4–0.5%) in females in 2015 (ESM_6).

Oman had the largest burden of age-standardized YLDs rate for other causes of vision loss and Lebanon had the smallest in both 1990 and 2015. Age-standardized YLDs were 34.9 (95% UI 24.7–47.6) per 100,000 males and 35.5 (95% UI 25.0–47.8) per 100,000 females in 1990, and 37.7 (95% UI 26.8–51.0) per 100,000 males and 38.6 (95% UI 27.2–51.8) per 100,000 females in 2015 (ESM_6).

### Age-specific prevalence of the leading causes of vision loss

**Fig. 3:**
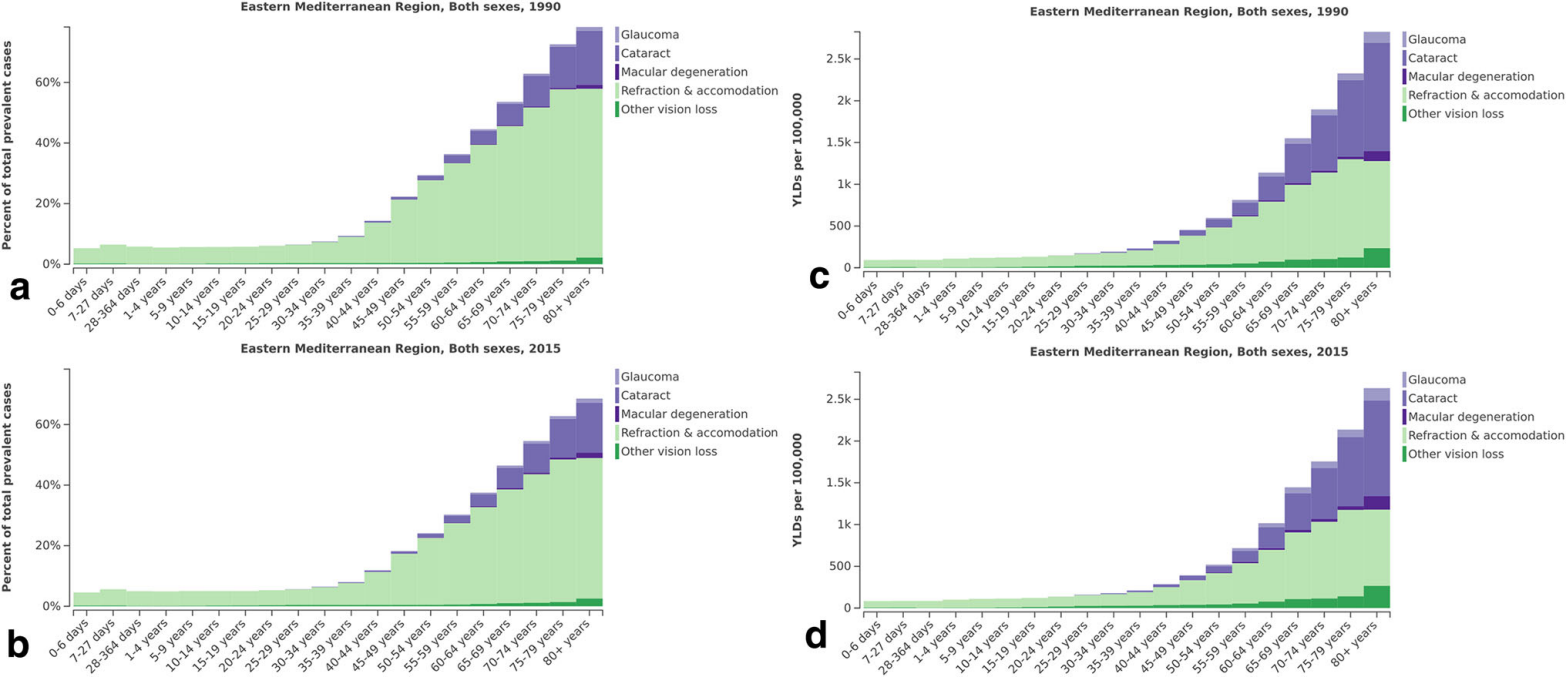
Age-specific prevalence (**a**, **b**) and years lived with disability (YLDs) rate (**c**, **d**) for causes of vision loss in Eastern Mediterranean Region at two time points (1990 and 2015) (Global Burden of Disease Study 2015, Eastern Mediterranean Region, 1990, 2015)

The main causes of vision loss, including cataract, glaucoma, macular degeneration, and the category “other causes of vision loss” were most prevalent in the population aged 80 and older in both 1990 and 2015. The highest prevalence of refraction and accommodation disorders was noted among people aged 70–74 years in 1990 and 2015 (Fig. 3).

### Leading causes of age-specific YLDs rate for vision loss

Figure 3 shows the highest YLDs rates for cataract, glaucoma, macular degeneration, and other causes of vision loss in the population aged 80 or older in 1990 and 2015. The highest rate of YLDs for refraction and accommodation disorders was demonstrated in the age group 70–74 years in 1990 and 2015 (Fig. 3).

### Association of SDI and changes in age-standardized prevalence and YLDs rate of vision loss

**Fig. 4:**
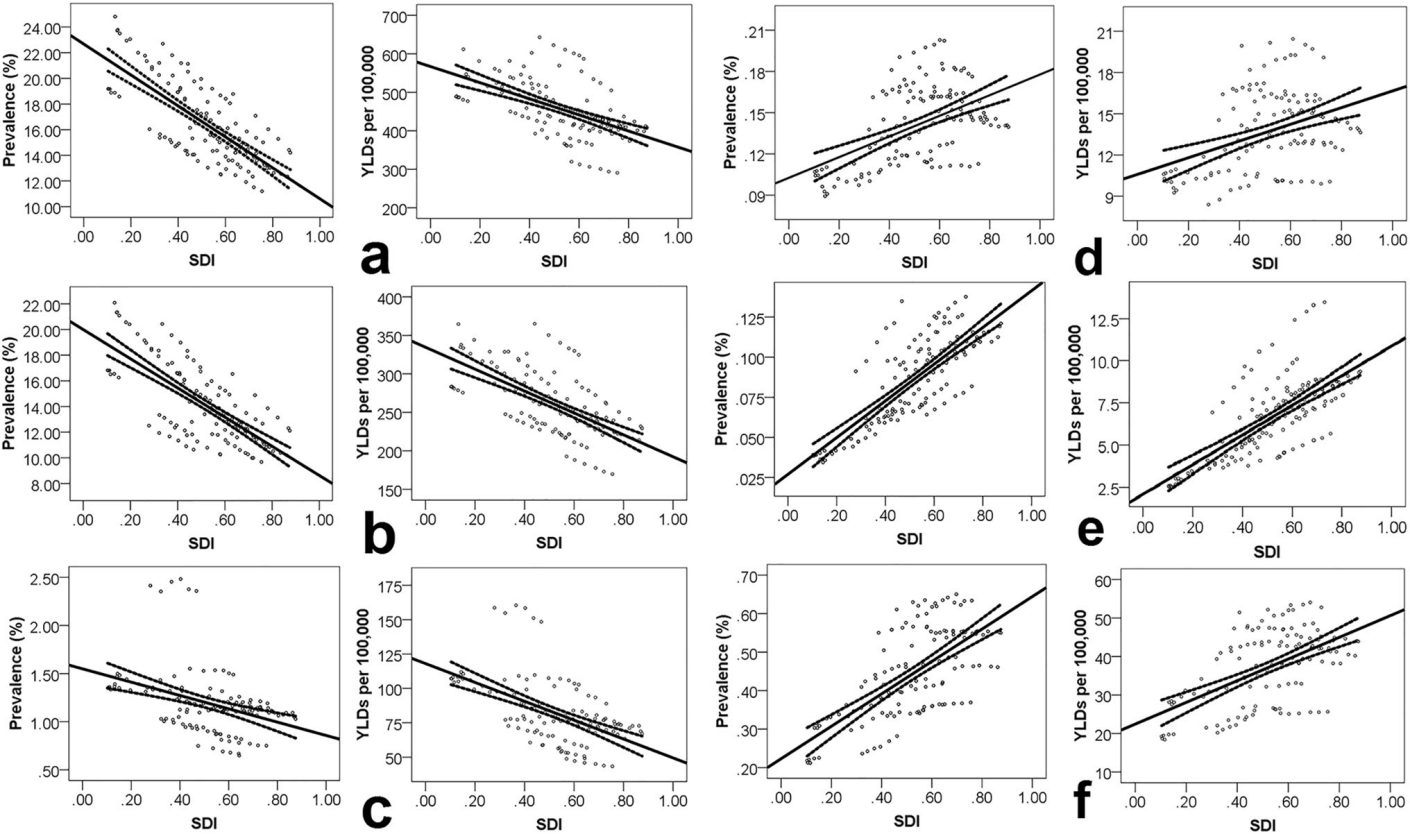
Association of Socio-demographic Index (SDI) and changes in age-standardized prevalence and years lived with disability (YLDs) rate of all-cause vision loss (**a**), refraction and accommodation disorders (**b**), cataract (**c**), glaucoma (**d**), macular degeneration (**e**), and other causes of vision loss (**f**) (Global Burden of Disease Study 2015, Eastern Mediterranean Region, 2015)

For each 0.1 unit increase in SDI, the age-standardized prevalence of vision loss due to all causes showed a 1.5% reduction using a multilevel linear model (*p* < 0.001). Corresponding values for each cause are presented in Table 2 and Fig. 4.

**Table 2:** Association of Socio-demographic Index and changes in age-standardized prevalence and years lived with disability rate of vision loss from 1990 to 2015 in the Eastern Mediterranean Region (Global Burden of Disease Study 2015, Eastern Mediterranean Region, 1990–2015)

Metric	Cause of vision loss	Estimate	95% UI		*p* value
			Lower	Upper	
Prevalence	All causes	−1.51	−1.59	−1.43	<0.001
	Refraction and accommodation disorders	−1.46	−1.53	−1.39	<0.001
	Cataract	−0.04	−0.05	−0.03	<0.001
	Glaucoma	0.00	0.00	0.01	<0.001
	Macular degeneration	0.01	0.01	0.01	<0.001
	Other vision loss	0.02	0.01	0.02	<0.001
YLDs	All causes	−23.94	−26.68	−21.20	<0.001
	Refraction and accommodation disorders	−17.89	−19.11	−16.67	<0.001
	Cataract	−4.41	−5.42	−3.40	<0.001
	Glaucoma	0.25	0.07	0.42	0.007
	Macular degeneration	0.90	0.81	0.98	<0.001
	Other vision loss	0.88	0.50	1.27	<0.001

*SDI* socio-demographic index, *UI* uncertainty interval, *YLDs* years lived with disability

For each 0.1 unit increase in SDI, a 23.9 per 100,000 population reduction in the age-standardized YLDs rate for vision loss due to all causes was noted using a multilevel linear model (*p* < 0.001). Cause-specific YLDs are shown in Table 2 and Fig. 4.

### Ratio of observed-to-expected prevalence and YLDs of vision loss based on SDI

ESM_7 shows that United Arab Emirates had the highest and Syria had the lowest observed-to-expected ratio (O/E) for prevalence of vision loss due to all causes based on SDI. The highest O/E YLDs ratio was noted in the Egypt, whereas Lebanon had the lowest ratio (ESM_8).

## Discussion

This is the first report on the burden of vision loss in the EMR countries during 1990–2015 (GBD 2015). Our findings indicated a decline in the age-standardized and age-specific prevalence and YLDs rate of vision loss from 1990 to 2015. However, vision loss still presents a burden in the region and needs to be addressed in health policies.

Our findings on trends were compatible with the results of the Khairallah et al. study, which showed a descending trend in age-standardized prevalence of blindness and moderate and severe VI in the Middle East and North Africa from 1990 to 2010 (Khairallah et al. 2014). Despite a declining trend in age-standardized prevalence and YLDs rate of vision loss over the past 25 years in the EMR, a wide disparity among the 22 countries in this region was demonstrated in terms of vision loss prevalence and YLDs rate. This can be explained by the difference in socio-demographic conditions and capacities of health systems in these countries (Mandil et al. 2013; Mokdad et al. 2014). On the other hand, in recent years, a number of EMR countries have been involved in conflicts and wars, resulting in limited health resources (Mokdad et al. 2016). Unfavorable and unstable socioeconomic conditions can also lead to a lack of strategic plans and operational programs for the prevention and treatment of VI and blindness.

The EMR had a higher age-standardized prevalence and YLDs rate of vision loss compared to the global rate and ranked third following the Southeast Asia and Africa regions in 2015. Stevens et al. reported a greater than 4% prevalence of blindness in older adults in Western sub-Saharan Africa, Eastern sub-Saharan Africa, the EMR, and South Asia in 2010 (Stevens et al. 2013). However, the highest reduction was observed in this region from 1990 to 2015 compared to all six world regions.

With regard to gender disparity, females were more affected by vision loss in the EMR, consistent with the global findings of the GBD 2015 study and other studies reporting on the EMR (Abou-Gareeb et al. 2001; GBD 2015; Hashemi et al. 2012; Jadoon et al. 2006; Khairallah et al. 2014; Rajavi et al. 2016; Stevens et al. 2013; WHO 2007). The gender disparity might be due to allocation of less family financial resources, resulting in limited access to eye care services for females (Hashemi et al. 2012; Stevens et al. 2013). This inequality may be attributed to cultural backgrounds which could be overcome by promoting awareness and education in affected societies.

Our results follow previous global and regional studies that reported refraction and accommodation disorders, cataract, glaucoma, and macular degeneration as the main causes of vision loss (GBD 2015; Khairallah et al. 2014; Katibeh et al. 2017a, b; Köberlein et al. 2013; Naidoo et al. 2014; Keeffe et al. 2014). Among these, refraction and accommodation disorders remained the most prevalent in the EMR from 1990 to 2015. A similar global-scale finding was reported in the GBD 2015 study and WHO global data 2010 (GBD 2015; Pascolini and Mariotti 2012; Bourne et al. 2013; Resnikoff et al. 2008; Naidoo et al. 2016). However, a decreasing trend in age-standardized prevalence and YLDs rate due to refraction and accommodation disorders was observed in this region. There was a 1.46% reduction in age-standardized prevalence and 17.89 per 100,000 population reduction in YLDs from refraction and accommodation disorders per 0.1 unit improvement in SDI score in the current study. Refraction and accommodation disorders had a considerable impact on the socioeconomic condition of the affected persons and their families via limiting educational and employment opportunities, resulting in productivity loss (Naidoo and Jaggernath 2012; Smith et al. 2009). A large amount of vision loss can be potentially prevented and cured through developing and implementing national screening programs and cost-effective interventions (WHO 2013).

Cataract was the second-ranked cause of vision loss in the EMR in both 1990 and 2015, with female predominance that was compatible with global findings from the GBD 2015 study (GBD 2015). The global WHO report also indicated that cataracts accounted for 33% of VI and 51% of blindness in 2010 (Pascolini and Mariotti 2012). Considering the geographic location of EMR countries, ultraviolet radiation may play a role in the high prevalence of cataract in this region (McCarty and Taylor 2002). Cataract can be treated simply with a timely and cost-effective intervention which would result in favorable visual outcomes; therefore it is categorized as a preventable cause of blindness (Pascolini and Mariotti 2012; WHO 2013). We found a significant correlation between the improvement of SDI score (by 0.1 unit) and the 0.04% reduction in age-standardized prevalence and 4.41 per 100,000 population reduction in the rate of YLDs from vision loss due to cataract in the EMR, which was in line with the Mundy et al. study. They reviewed the literature to report the association of cataract care with socioeconomic parameters in both developed and developing countries. These parameters can lead to limited access to primary eye care services. Promotion of education can improve the acceptability of these resources in low socioeconomic areas (Mundy et al. 2016).

Glaucoma was the third-leading cause of vision loss in the EMR. A slightly ascending trend of age-standardized prevalence and the rate of YLDs from glaucoma were observed over our study period. This finding follows the global results in the GBD 2015 study (GBD 2015). A meta-analysis by Bourne et al. showed an increased percentage of blindness due to glaucoma from 1990 to 2010 globally, with no significant difference between regions (Bourne et al. 2016). We also found that the slightly increasing age-standardized YLDs rate of glaucoma (0.25 per 100,000 person) was associated with the improved SDI score (by 0.1 unit). Given the increasing trend of vision loss due to glaucoma, a number of issues should be considered for public health planning. First, glaucoma and macular degeneration are responsible for the majority of irreversible blindness in the world. One out of 15 cases of blindness and one out of 45 cases of VI were due to glaucoma by 2010 (Bourne et al. 2016). Early diagnosis and proper medical and surgical management can prevent blindness due to glaucoma. Secondly, the Bourne et al. study also revealed that regions with younger populations had lower percentages of blindness due to glaucoma compared to high-income regions with older populations (Bourne et al. 2016). Tham et al. demonstrated the highest prevalence of glaucoma in Africa (primary open-angle glaucoma 4.20%) and Asia (primary angle-closure glaucoma 1.20%) in 2013 (Tham et al. 2014). Therefore, considering increasing life expectancy, the prevalence of VI due to glaucoma is expected to increase in the EMR in the future. Clinical and targeted screening would be appropriate approaches for preventing vision loss due to glaucoma (Mohammadi et al. 2014).

Macular degeneration was the fourth-leading cause of vision loss in terms of prevalence and YLDs rate in the EMR. An increasing trend was observed in age-standardized prevalence and rate of YLDs due to macular degeneration in both sexes. Our finding is consistent with the global results from GBD 2015 and the meta-analysis from 1990 to 2010 (GBD 2015; Jonas et al. 2014). This meta-analysis demonstrated a lower prevalence of macular degeneration in regions with younger populations in comparison with high-income regions (Jonas et al. 2014). Our study also showed that the increase in age-standardized prevalence (0.01%) and YLDs rate (0.9 per 100,000 person) of macular degeneration from 1990 to 2015 was associated with the improvement of SDI score (by 0.1 unit). With regard to the aging population and availability of effective interventions, especially intravitreal anti-vascular endothelial growth factor drugs, macular degeneration may be recognized as an important public eye health issue for future planning (Jonas et al. 2014).

Our study has a few limitations. Vision loss due to diabetes mellitus is considered part of the diabetes burden and is not included in our study (Moradi-Lakeh et al. 2017). Considering the high prevalence of diabetes in EMR, the burden of VI may be higher than what is estimated in this study (Katibeh et al. 2017a, b; Khandekar 2012; WHO 2016). Some of the countries in the region do not have appropriate data on the epidemiology of low vision or have used nonstandard methods for measuring and reporting vision loss. We used the GBD general methodology to produce more accurate estimates using different study-level or country-level covariates.

### Conclusions

The current study provides an up-to-date estimation based on the GBD 2015 study, demonstrating a high prevalence and high rate of YLDs due to vision loss with a decreasing trend in the EMR. Our findings call for developing and implementing programs to manage refraction and accommodation disorders and cataract due to their large burden. There is a need for balance between prevention and treatment programs to reduce the burden of vision loss in the region. This can be achieved by developing and implementing a national operational program and involving all related stakeholders. Education campaigns might be useful to promote public awareness.

## Electronic supplementary material

Below is the link to the electronic supplementary material.

Supplementary material 1 (XLSX 21 kb)

Supplementary material 2 (DOCX 4631 kb)
