# Supplementary material for: Burden of vision loss in the Eastern Mediterranean region, 1990–2015: findings from the Global Burden of Disease 2015 study
Source: Int J Public Health. 2017 Aug 3;63(Suppl 1):199–210. doi: 10.1007/s00038-017-1000-7 (PMC5973987; doi:10.1007/s00038-017-1000-7)
Supplement: Supplementary file 2 — Supplementary material 2 (DOCX 4631 kb) [file 38_2017_1000_MOESM2_ESM.docx]

Electronic Supplementary Material

**Article title:**

Burden of vision loss in the Eastern Mediterranean region, 1990–2015: findings from the Global Burden of Disease 2015 study

**Journal:**

International Journal of Public Health

**Authors:**

GBD 2015 Eastern Mediterranean Region Vision Loss Collaborators

**Corresponding author:**

Ali H. Mokdad

Institute for Health Metrics and Evaluation, University of Washington, Seattle, WA, United States

Email: [mokdaa@uw.edu](mailto:mokdaa@uw.edu)

**Electronic Supplementary Material 1:** Age-standardized prevalence and years lived with disability rate of vision loss in the world regions in 1990 and 2015. (Global Burden of Disease Study 2015, WHO world regions, 1990 and 2015)

|  | 1990 | | | 2015 | | |  | Percent  of  change |  | 1990 | | | 2015 | | |  | Percent  of  change |
| --- | --- | --- | --- | --- | --- | --- | --- | --- | --- | --- | --- | --- | --- | --- | --- | --- | --- |
| **World regions** | Prevalence (%) | 95% UI | | Prevalence (%) | 95% UI | |  |  |  | YLDs rate per 100,000 persons | 95% UI | | YLDs rate per 100,000 persons | 95% UI | |  |  |
|  |  | Lower | Upper |  | Lower | Upper |  |  |  |  | Lower | Upper |  | Lower | Upper |  |  |
| African Region | 19.5 | 18.5 | 20.5 | 17.6 | 16.8 | 18.5 |  | -9.41 |  | 518.3 | 361.9 | 722.9 | 490.6 | 344.9 | 681.9 |  | -5.34 |
| Eastern Mediterranean Region | 18.2 | 17.5 | 19.0 | 15.5 | 14.8 | 16.2 |  | -15.09 |  | 536.9 | 378.5 | 746.0 | 482.3 | 342.5 | 667.8 |  | -10.16 |
| European Region | 10.8 | 10.4 | 11.2 | 9.8 | 9.4 | 10.1 |  | -9.86 |  | 245.2 | 166.3 | 358.2 | 231.8 | 159.1 | 334.7 |  | -5.49 |
| Region of the Americas | 10.6 | 10.2 | 10.9 | 10.8 | 10.5 | 11.2 |  | 2.38 |  | 243.0 | 166.3 | 355.0 | 254.5 | 174.6 | 368.6 |  | 4.71 |
| South-East Asia Region | 19.8 | 19.0 | 20.5 | 18.5 | 17.7 | 19.3 |  | -6.34 |  | 558.9 | 391.1 | 782.3 | 529.5 | 372.8 | 739.1 |  | -5.26 |
| Western Pacific Region | 11.7 | 11.3 | 12.1 | 11.9 | 11.5 | 12.4 |  | 1.82 |  | 282.2 | 195.5 | 404.9 | 295.2 | 203.3 | 420.8 |  | 4.57 |
| Global | 13.6 | 13.1 | 14.1 | 13.4 | 12.9 | 13.9 |  | -1.32 |  | 344.5 | 238.8 | 488.1 | 352.7 | 245.3 | 494.9 |  | 2.39 |

UI, uncertainty interval; YLDs, years lived with disability


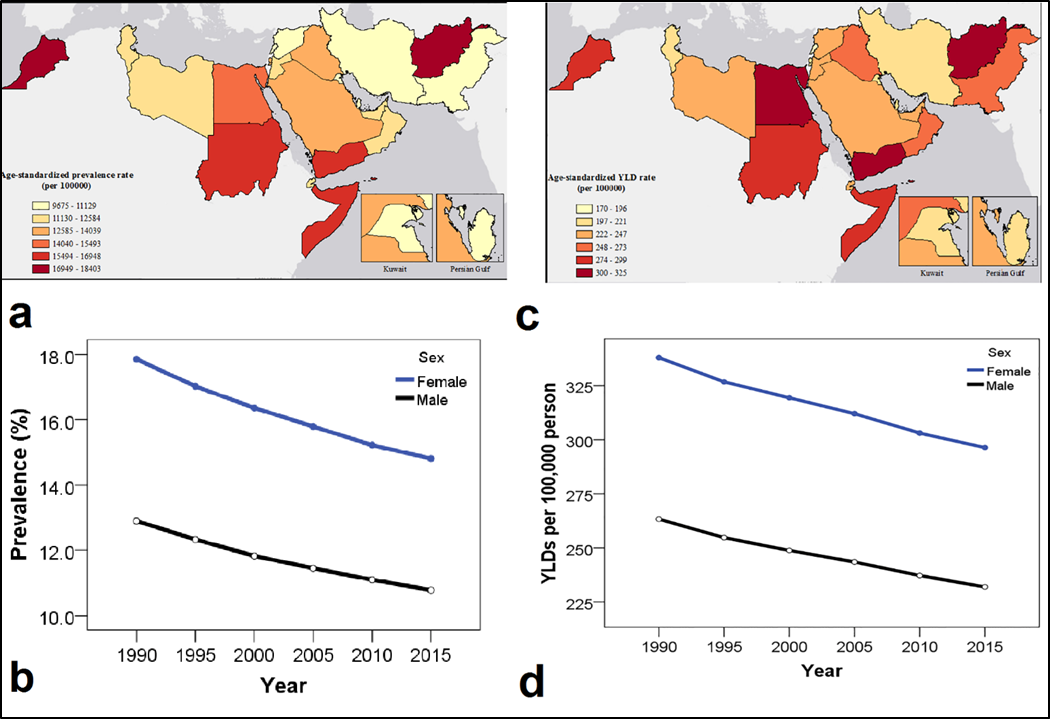


**Electronic Supplementary Material 2:** Age-standardized prevalence of refraction and accommodation disorders in the Eastern Mediterranean Region by countries, both sexes, in 2015 (a), trend in age-standardized prevalence of refraction and accommodation disorders in the Eastern Mediterranean Region by sex from 1990 to 2015 (b), age-standardized years lived with disability (YLDs) per 100,000 persons for refraction and accommodation disorders in the Eastern Mediterranean Region by countries, both sexes in 2015 (c), trend in age-standardized years lived with disability per 100,000 persons for refraction and accommodation disorders in the Eastern Mediterranean Region by sex from 1990 to 2015 (d). (Global Burden of Disease Study 2015, Eastern Mediterranean Region, 1990 - 2015)


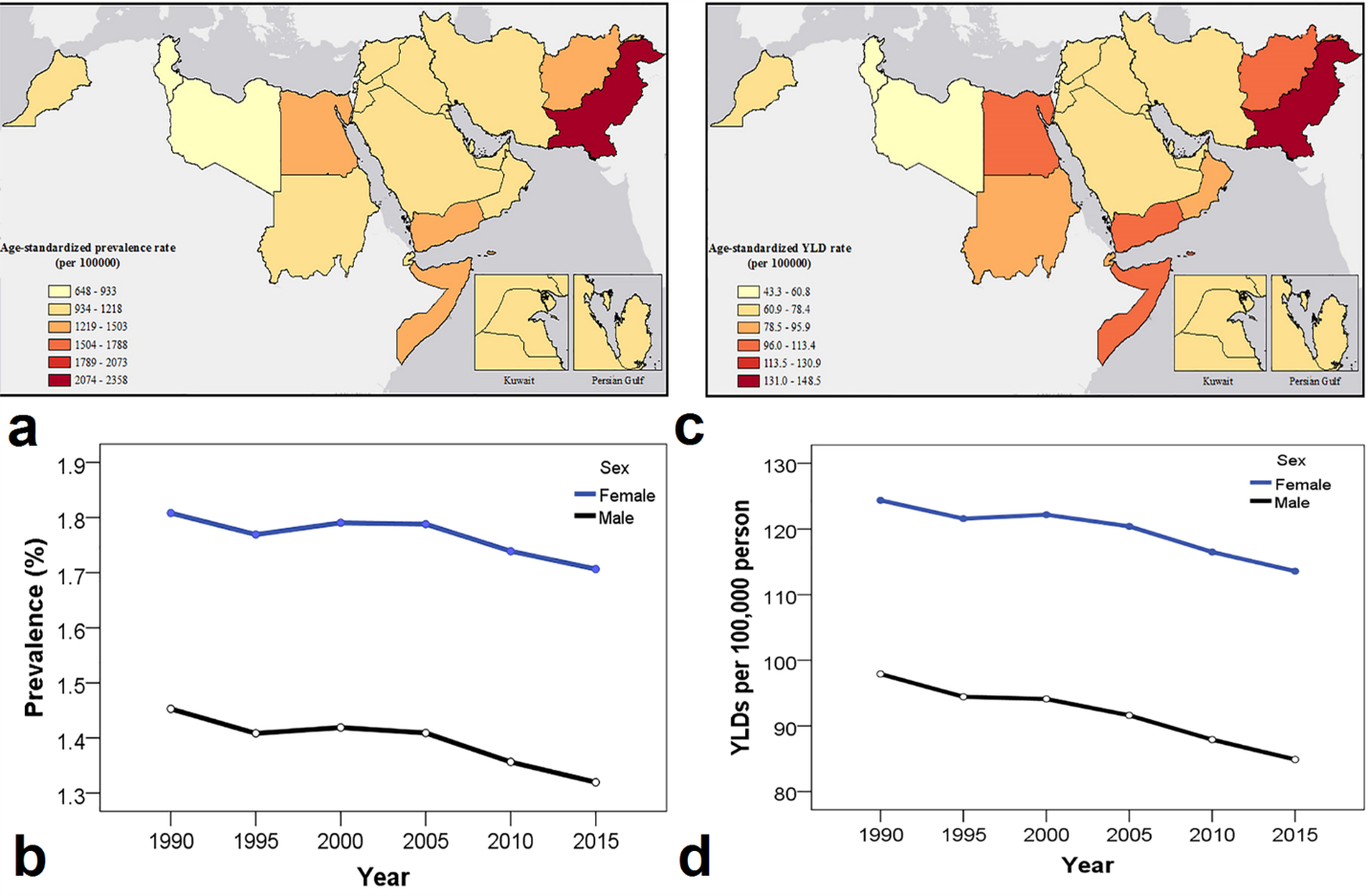


**Electronic Supplementary Material 3:** Age-standardized prevalence of cataract in the Eastern Mediterranean Region by countries, both sexes, in 2015 (a), trend in age-standardized prevalence of cataract in the Eastern Mediterranean Region by sex from 1990 to 2015 (b), age-standardized years lived with disability(YLDs) per 100,000 persons for cataract in the Eastern Mediterranean Region by countries, both sexes, in 2015 (c), trend in age-standardized years lived with disability per 100,000 persons for cataract in the Eastern Mediterranean Region by sex from 1990 to 2015 (d) (Global Burden of Disease Study 2015, Eastern Mediterranean Region, 1990 - 2015)


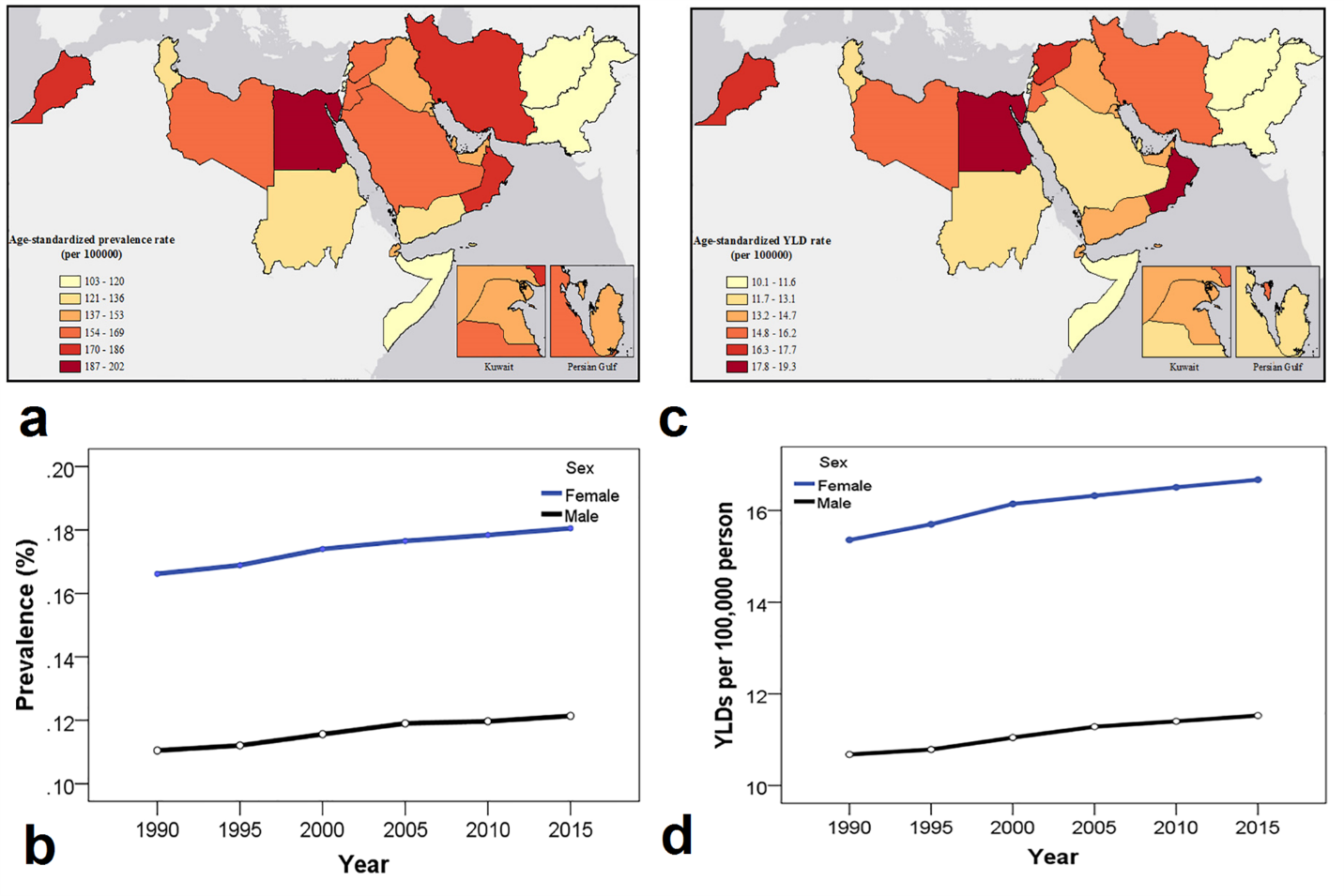


**Electronic Supplementary Material 4:** Age-standardized prevalence of glaucoma in the Eastern Mediterranean Region by countries, both sexes, in 2015 (a), trend in age-standardized prevalence of glaucoma in the Eastern Mediterranean Region by sex from 1990 to 2015 (b), age-standardized years lived with disability per 100,000 persons for glaucoma in the Eastern Mediterranean Region by countries, both sexes, in 2015 (c), trend in age-standardized years lived with disability(YLDs) per 100,000 persons for glaucoma in the Eastern Mediterranean Region by sex from 1990 to 2015 (d) (Global Burden of Disease Study 2015, Eastern Mediterranean Region, 1990 - 2015)


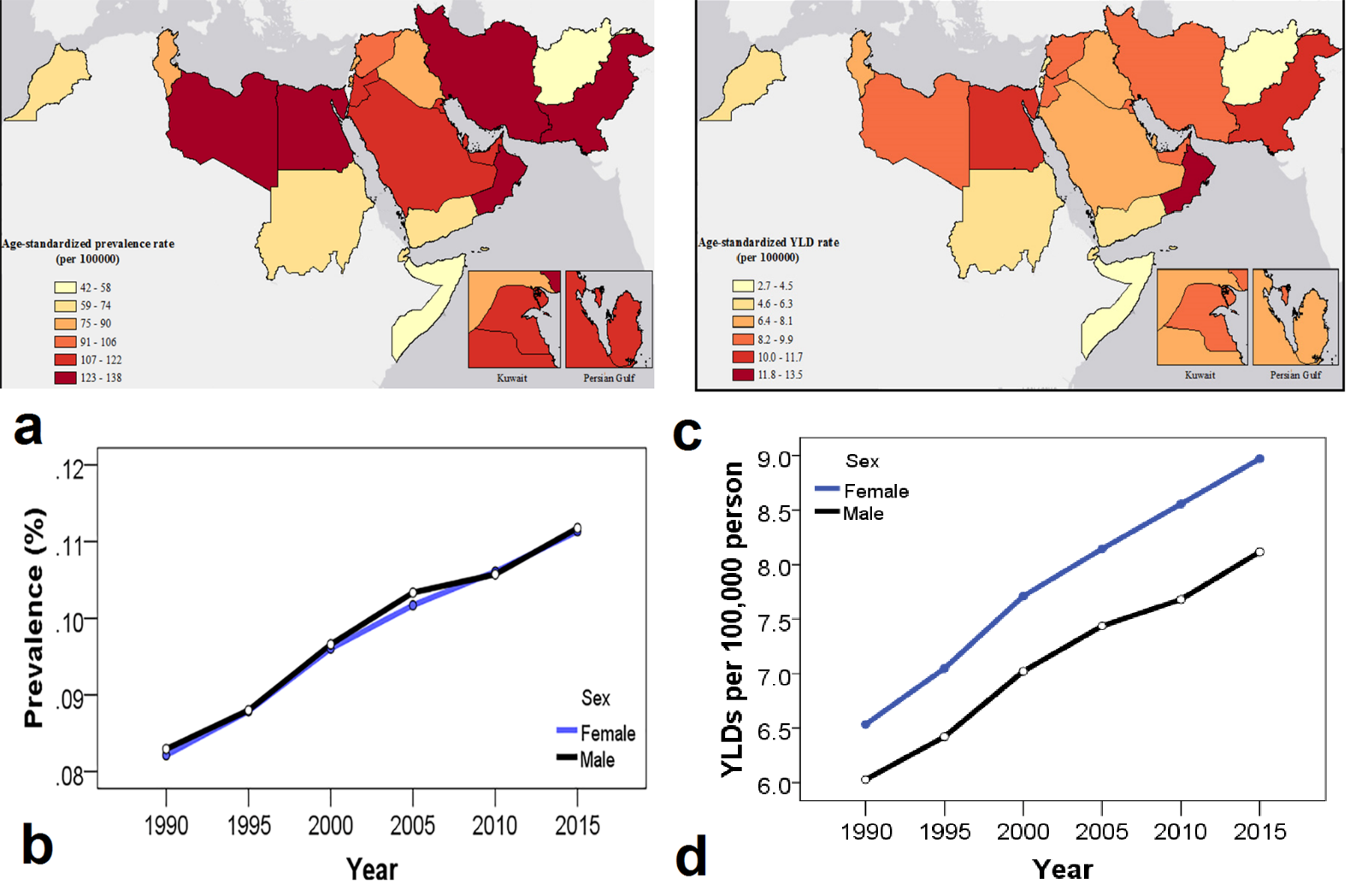


**Electronic Supplementary Material 5:** Age-standardized prevalence of macular degeneration in the Eastern Mediterranean Region by countries, both sexes, in 2015 (a), trend in age-standardized prevalence of macular degeneration in the Eastern Mediterranean Region by sex from 1990 to 2015 (b), age-standardized years lived with disability per 100,000 persons for macular degeneration in the Eastern Mediterranean Region by countries, both sexes, in 2015 (c), trend in age-standardized years lived with disability(YLDs) per 100,000 persons for macular degeneration in the Eastern Mediterranean Region by sex from 1990 to 2015 (d) (Global Burden of Disease Study 2015, Eastern Mediterranean Region, 1990 - 2015)


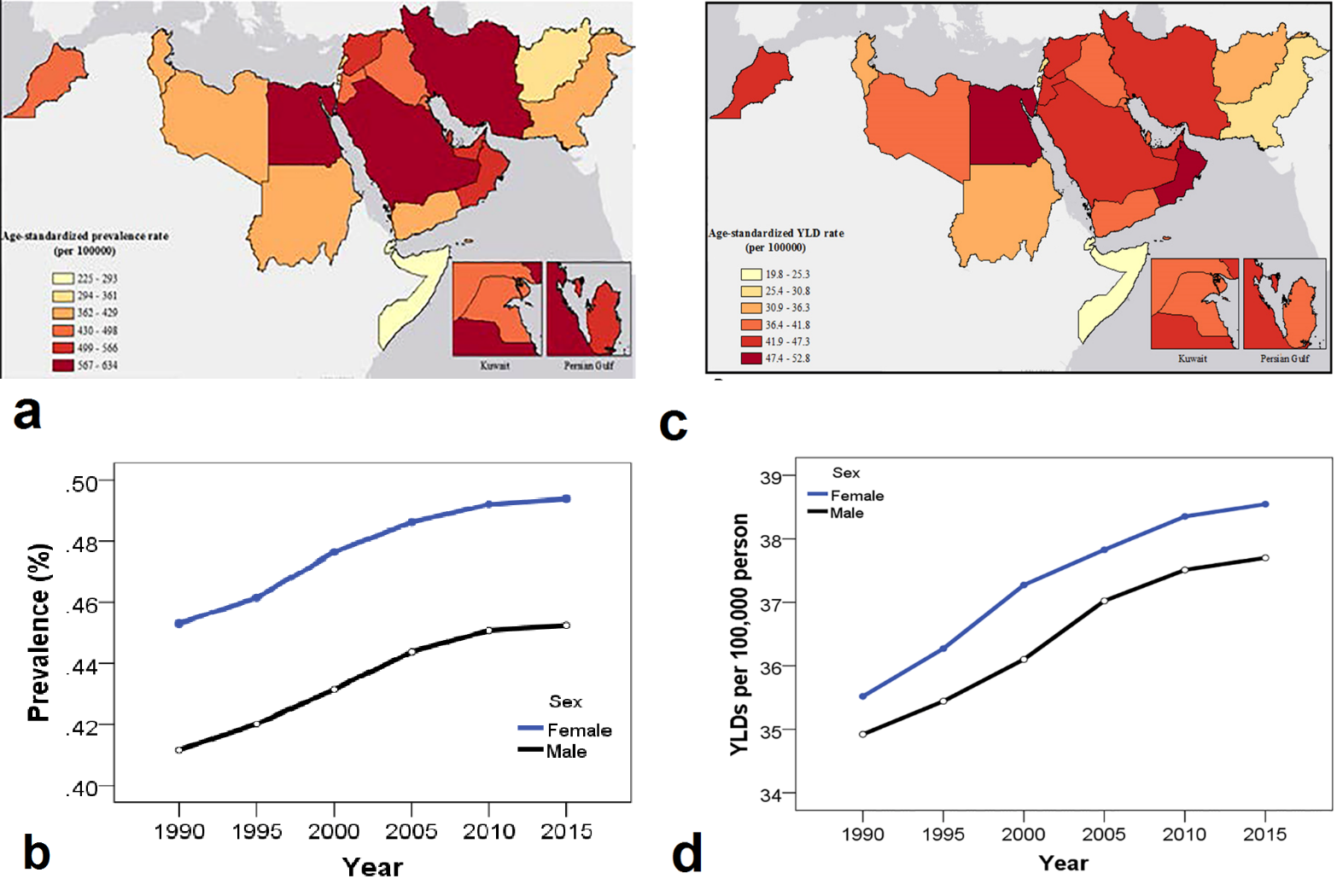


**Electronic Supplementary Material 6:** Age-standardized prevalence of other causes of vision loss in the Eastern Mediterranean Region by countries, both sexes, in 2015 (a), trend in age-standardized prevalence of other causes of vision loss in the Eastern Mediterranean Region by sex from 1990 to 2015 (b), age-standardized years lived with disability per 100,000 persons for other causes of vision loss in the Eastern Mediterranean Region by countries, both sexes, in 2015 (c), trend in age-standardized years lived with disability(YLDs) per 100,000 person for other causes of vision loss in the Eastern Mediterranean Region by sex from 1990 to 2015 (d) (Global Burden of Disease Study 2015, Eastern Mediterranean Region, 1990 - 2015)

**
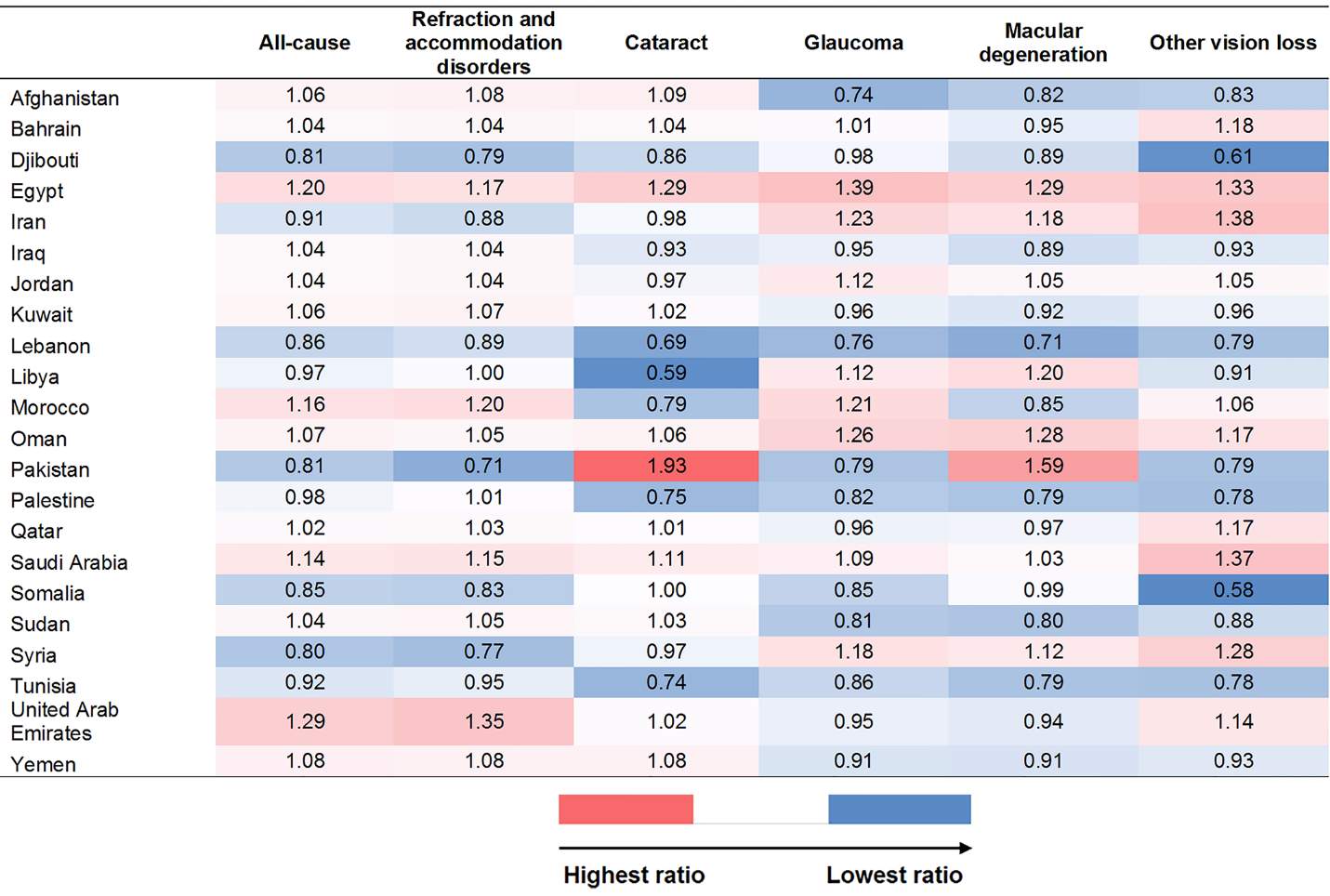
**

**Electronic Supplementary Material 7:** Ratio of observed to expected prevalence of all-cause and cause-specific vision loss based on Socio-demographic Index(SDI) in Eastern Mediterranean Region countries (Global Burden of Disease Study 2015, Eastern Mediterranean Countries, 2015)


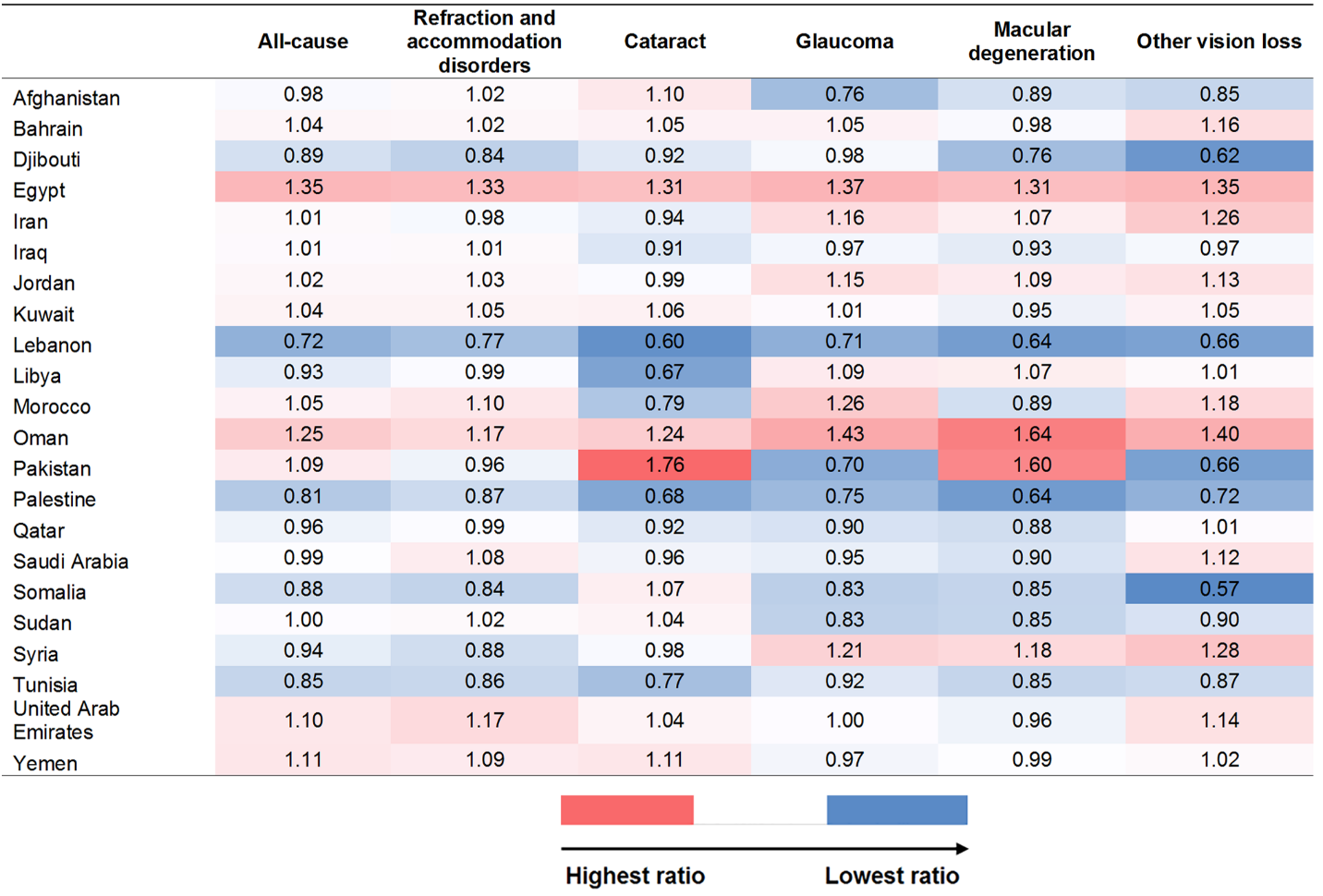


**Electronic Supplementary Material 8:** Ratio of observed to expected years lived with disability(YLDs) for all-cause and cause-specific vision loss based on Socio-demographic Index(SDI) in Eastern Mediterranean Region countries (Global Burden of Disease Study 2015, Eastern Mediterranean Countries, 2015)
